# Rainbows in my ears—Synesthetic color perception with partial-reduced and morphed musical instrument timbres

**DOI:** 10.3389/fpsyg.2025.1697918

**Published:** 2025-11-19

**Authors:** Christoph Reuter, Isabella Czedik-Eysenberg, Sarah Ambros, Saleh Siddiq, Solange Glasser, Jamie Ward, Jörg Jewanski

**Affiliations:** 1 Space for Interdisciplinary Experiments on Sound (SInES), University of Vienna, Vienna, Austria; 2 Melbourne Conservatorium of Music, University of Melbourne, Melbourne, VIC, Australia; 3 School of Psychology, University of Sussex, Brighton, United Kingdom

**Keywords:** synesthesia, timbre-induced color perception, partials, sound morphing, audio signal analysis

## Abstract

**Background:**

Synesthesia is a perceptual phenomenon in which sensory inputs automatically elicit consistent additional sensations. Sound-color synesthesia, one of the most widely recognized forms, involves experiencing colors and shapes in response to auditory stimuli. Within this, timbre-color synesthesia, reported in 26% of music-color synesthetes, has rarely been studied in depth, particularly regarding how specific timbre manipulations influence color perception.

**Methods:**

This study investigated whether changes in timbre-induced color perception occur continuously or abruptly as sound parameters are altered, and whether generalizable patterns can be identified. For the first time, Δ*E* color similarity calculations were applied to objectively assess participants' timbre-color synesthesia. Participants listened to loudness-matched single tones (flute, oboe, French horn, violin, and piano) from the Vienna Symphonic Library, presented both in their original form and with manipulated timbres. Manipulations included partial reduction (removing partials from the first to the tenth) and morphing between instrument pairs across seven stages (from 100:0% to 0:100%). After each sound, participants described their color perception using a color selection field, generating RGB and HSL values of the chosen colors.

**Results:**

Findings revealed correlations between the individual color perceptions. Increasing partial reduction was associated with decreased saturation and increased lightness, particularly for flute, oboe, violin, and piano. Using audio features such as spectral centroid, harmonicity strength, and percussive loudness, generalizable rules for the perception of timbre-induced colors in timbre-color synesthetes could be observed. For morphed sounds, perceived colors shifted progressively from those associated with the initial instrument toward those associated with the target instrument across the morphing stages. Here, too, spectral centroid, harmonicity strength, and percussive loudness showed strong overall correlations between instrumental sounds and the colors they induce.

**Conclusion:**

This study demonstrates that manipulating musical instrument timbre through partial reduction and morphing induce clear but individual patterns of color perception in timbre-color synesthetes, which differ significantly from the responses of non-synesthetes.

## 1 Introduction

Synesthesia is a fascinating perceptual phenomenon in which sensory input or cognitive concepts (such as units of measurement or the alphabet) from one sense or sense modality automatically elicit additional, consistent sensations in a second sense or sense modality (Simner and Hubbard, 2013). The prevalence of synesthesia is estimated at 4%, with an equal female-to-male ratio (Simner et al., 2006). Synesthetic experiences show a consistency rate of 70–90% (Asher et al., 2006). The most commonly reported concurrent sensations are visual, occurring in 97% of cases, followed by spatial configurations at 62% (Niccolai et al., 2012). To date, at least 112 distinct types of synesthesia have been identified (Ward and Simner, 2022). Among these, sound-color synesthesia is one of the most widely recognized, in which auditory stimuli evoke the perception of colors and shapes. The prevalence of this type among all forms of synesthesia remains unclear, with estimates ranging from 18.7% (Barnett et al., 2008) to 41% (Niccolai et al., 2012).

It is also unclear how many types of auditory inputs are involved in sound-related forms of synesthesia. The first documented case, dating back to 1812, describes a synesthete who experienced three forms of music-color synesthesia: tones, intervals, and timbres of musical instruments (Jewanski et al., 2009). Overviews cataloging possible auditory inputs for music-color synesthesia have appeared since 1881 and continue to the present day, yet no consensus has been reached regarding their classification. The most frequently cited inducers include pitch height, timbre, chord, key, and complete musical piece (Jewanski, 2025). Among these, timbre is mentioned in nearly every overview.

Studies focusing on timbre-color synesthesia are relatively rare. Beyond case studies that provide detailed analyses of one or two individuals (Mills et al., 2003; Fernay et al., 2012; Farina et al., 2017; Zdzinski et al., 2019; Bartuliene et al., 2025), empirical investigations have examined how color associations vary according to timbre. These studies have compared perceptual responses across different instruments and sound types, including piano, string instruments, and sinus tones (Ward et al., 2006, 2025); flute, piano, and violin (Chiou et al., 2013); 17 instruments (Griscom, 2014, pp. 65–78); 16 instruments (Griscom and Palmer, 2014); and 14 instruments (Menouti et al., 2015). Other work has explored how synesthetic color associations differ when the same pieces of music are performed on a participant's principal instrument, on an unfamiliar instrument, or produced electronically (Curwen et al., 2024).

Music-color synesthesia appears to influence artistic and musical creativity more profoundly than other forms of synesthesia [for art: Lunke and Meier (2019); for music: Glasser (2018, 2022); for synesthesia and creativity: Mulvenna (2013)]. A number of musicians have reported experiencing music-color synesthesia, or have been described as synesthetes, across both classical and popular music traditions. Within classical music, examples include Amy Beach (Logan, 2015; Clarke, 2022), György Ligeti (Engelbrecht et al., 1997; Noeske, 2016), Olivier Messiaen (Jewanski, 2006; Glasser, 2016), and Michael Torke (Ruch-Appold, 1999; Duffy, 2001, pp. 79–108; Sacks, 2007, pp. 168–171). Comparative studies have also examined Aleksandr Skryabin, Alexander László, Olivier Messiaen, and Michael Denhoff (Jewanski, 2002). Beyond the classical domain, music-color synesthesia has likewise been documented among pop, rock, and jazz musicians (Day and Jewanski, 2020).

Visual artists have also been influenced by music-color synesthesia (for David Hockney: Cytowic, 1989, pp. 28 and 274–283; for Wassily Kandinsky: Ione and Tyler, 2003; Just, 2017; for Paul Klee: Ione, 2004; Schawelka, 2006). Art students demonstrate a higher prevalence of synesthesia (Rothen and Meier, 2010), and synesthetes more frequently engage in artistic practice (Ward et al., 2008; Lunke and Meier, 2022). The exhibition *Synesthesia: Art and the Mind* was entirely devoted to artists known to be synesthetes (David Hockney, Joan Mitchell, Marcia Smillack, and Carol Steen) or believed to have been synesthetes (Charles Burchfield, Tom Thomson, Wassily Kandinsky, and Vincent van Gogh) (Berman and Steen, 2008; see also Cavallaro, 2013). Other exhibitions, such as *Visual Music: Synaesthesia in Art Music since 1900* (Hyun, 2005) and *Sensory Crossovers: Synesthesia in American Art* (Udall and Weekly, 2010) do not clearly distinguish between synesthetic, associative, symbolic, and metaphoric connections across the arts.

Given that music-color synesthesia can influence both musicians and visual artists, and that timbre-color synesthesia occurs with a frequency of 26% within this form of synesthesia (Niccolai et al., 2012), it is well justified to investigate timbre-color synesthesia in greater detail. To date, however, no studies have examined differences in timbre within a single instrument, even though variations such as high, middle, and low registers can elicit distinct color perceptions in synesthetes (Siddiq et al., 2024).

## 2 Research question

In the light of this background, it is important to investigate how timbre-induced color perception in synesthetes changes when the spectral properties of musical instruments are systematically altered. The present study therefore aims to examine how manipulations of timbre—through the stepwise reduction of partials (from the first to the tenth partial) and through seven-step morphing between different instrument sounds—affect the color perceptions of timbre-color synesthetes.

Accordingly, our research questions are as follows:

1. How does manipulating sounds by reducing their partial structure or by morphing their timbres affect the color perceptions of synesthetes?
2. Does the change in induced color perception occur continuously or abruptly?
3. Are there identifiable typical patterns in these perceptual changes?

## 3 Methods

An online study was conducted with 20 timbre-color synesthetes (5 m, 15 f, aged from 21 to 72, Ø 41, SD: 16.9) recruited via the database of synesthetes at the School of Psychology, University of Sussex and German Synesthesia Association [“Deutsche Synästhesie-Gesellschaft (DSG)”]. The test subjects listened to loudness-matched individual sounds of flute, oboe, horn, violin and piano from the Vienna Symphonic Library (played on C4 in *mf* , 2 s duration) and their alterations presented in a randomly mixed order: Morphed sounds between two of the musical instrument timbres in the morph-ratios 100:0%, 90:10%, 70:30%, 50:50%, 30:70%, 10:90% and 0:100% between the respective sounds as well as partial-reduced variants of the five instrument timbres in which the lowest 10, 9, 8 etc. to 1 partial(s) in the spectrum were removed. The timbres have been morphed in different ratios with the VST plug-in MMorph from MeldaProductions; the partials have been removed with the spectral editor in Adobe Audition. The original sounds of the five musical instruments were played twice in random order to check whether the same sounds induced the same color perception in the synesthetes (= intra-rater reliability). The task of the test subjects was to select the timbre-induced color perception in a color selection field after each sound was heard, so that at the end of the study a corresponding color value in HSL (hue (chroma), saturation and lightness) was available for each sound.

### 3.1 Fantastic timbre-color synesthetes and how to find them

Rather than perceiving simple and discrete colors such as “blue” or “red”, music-color synesthetes (and by extension timbre-color synesthetes) often describe their experience of music in more complex and nuanced terms, for example, as “shades of dark orangish red, like embers glowing in a fireplace” (Siddiq et al., 2024, p. 185). Their perceptions often extend beyond two-dimensional descriptions, such as a “green background with white in the foreground” (Cytowic, 2018, p. 138) and can combine colors with shapes and textures: “Surfaces have a fine or very torn structure depending on the character of the sound” (Waldeck, 2006, p. 111). Thus, it cannot be expected that the colors documented by the test subjects correspond to precise HSL values. If the same sound is heard a second time, it cannot be assumed that the color selection induced will absolutely match the color values of the first sound (Simner, 2012). Some degree of fluctuation in the results is inevitable, and this raises the question of the range within which hue (H), saturation (S), and lightness (L) must be maintained for the color associated with the same sound to still be considered identical or sufficiently similar. In other words: how much variation in induced colors is permissible before timbre-color synesthesia can no longer be classified?

The CIEDE2000 algorithm (Cui et al., 2001) offers a way to calculate the similarity of two colors on a scale from 0 Δ*E* (i.e., colors are exactly the same) to 100 Δ*E* (i.e., colors are exactly the opposite) based on human color perception. According to this algorithm, a just noticeable difference between two colors in an AB comparison is at about 1 Δ*E*. As the test subjects in our experiment had neither a color AB comparison nor saw real colors, but rather dynamic color patterns induced by timbres, the similarity of the perceived colors can be assumed to be at larger values. Here, the Lightfastness Category III as stated in the ASTM D4303 standard [American Society for Testing and Materials (ASTM), 2022] would be suitable for calculation. This category states that colors with a distance of 8 to 16 Δ*E* can be distinguished but still produce a similar color impression. With a color difference greater than 16 Δ*E*, the point is reached where two colors can be considered as different (like green vs. yellow, red vs. orange, blue vs. violet, etc.). Or to put it in other words: colors with a color difference of 16 or more Δ*E* are so different that they are no longer the same. Even below 16 Δ*E*, color differences are clearly noticeable, but the colors would still be considered as similar (like e.g., a bright yellow vs. a dull yellow).

This algorithm can be used to determine the accuracy with which the sound-induced color synesthesias are matched for the same instrument sounds. From the sum of the similarities achieved (in Δ*E*) for all five original timbres in their repetitions (C4 played on flute, violin, French horn, piano and oboe) divided by the number of responses from the test subjects, both a score for the degree of timbre color synesthesia can be determined and a limit can be found above which timbre color synesthesia can no longer be assumed (see Table 1).

**Table 1:** Similarity of perceived colors in Δ*E* for the same sounds per test subject (case no.) as well as the mean Δ*E* (last column) as an indicator of the degree of timbre color synesthesia (**green**: < 8–10 Δ*E*, **yellow**: 10–13 Δ*E*, **red**: 13–16 Δ*E*).

**Case no**.	**Flute Δ*E***	**Violin Δ*E***	**Horns Δ*E***	**Piano Δ*E***	**Oboe Δ*E***	**Total Δ*E***	**Mean Δ*E***
**562**	13.28	16.58	19.67	7.16	8.77	65.46	13.092
**581**	10.95	8.57	8.61	6.78	21.64	56.55	11.31
**584**	55.43	50.73	4.9	50.26	33.89	195.21	39.042
**590**	2.22	79.3	35.21	40.63	20.55	177.91	35.582
**594**	48.48	2.42	6.39	10.33	1.66	69.28	13.856
**596**	8.00	9.33	6.61	14.41	58.44	96.79	19.358
**599**	31.61	8.36	38.15	46.21	5.43	129.76	25.952
**601**	8.37	3.92	15.43	14.52	25.71	67.95	13.59
**604**	38.95	12.39	3.35	5	0.93	60.62	12.124
**609**	0.35	16.06	NaN	0.86	25.71	42.98	10.745
**614**	31.59	52.72	22.86	3.29	17.6	128.06	25.612
**617**	15.11	33.96	8.77	10.42	5.06	73.32	14.664
**621**	47.14	33.22	33.37	17.62	56.15	187.5	37.5
**623**	23.67	62.1	23.55	7.53	42.96	159.81	31.962
**624**	41.11	78.57	52.68	15.01	72.79	260.16	52.032
**626**	26.92	11.88	8.65	2.19	5.09	54.73	10.946
**629**	8.86	3.9	12.85	2.86	12.69	41.16	8.232
**635**	13.26	47.37	46.32	5.05	41.64	153.64	30.728
**640**	5.79	10.58	8.97	0.96	10.18	36.48	7.296
**643**	1.31	48.32	5.78	40.3	8.33	104.04	20.808

< 8–10 ΔE: degree of timbre color synesthesia: excellent.

10–13 ΔE: degree of timbre color synesthesia: very well.

13–16 ΔE: degree of timbre color synesthesia: satisfactory.

>16 ΔE: degree of timbre color synesthesia: poor (i.e., no timbre color synesthesia).

The result clearly shows that only half of the recruited timbre color synesthetes were able to demonstrate the corresponding skills. Thus, for further evaluation, a group of 10 timbre color synesthetes (2 excellent, 4 very well and 4 satisfactory) can be compared to a group of 10 non-synesthetes.^1^

Subsequently, we used correlation analyses to find out whether a connection between the number of reduced partials or the morphing ratio between two timbres and the sound-induced color perception for the group of synesthetes, non-synesthetes or both groups (all) was discernible. Additionally, all timbres were analyzed for their audio features with the help of PADMEA (Czedik-Eysenberg, 2021), focusing on specific audio features extracted via the signal analysis libraries Librosa (McFee et al., 2015) and Essentia (Bogdanov et al., 2013). The results of this audio signal analysis were also checked for correlations with the color values (RGB and HSL) of the induced color perceptions.

## 4 Results

### 4.1 Timbre-induced color perception with partial-reduced sounds

Given the individuality of synesthetic color perception and the different manifestations of timbre-color synesthesia among the test subjects described above, it would be surprising if correlations could be found between all instruments and all sound-induced color perceptions. However, clear interrelationships can be identified between some instrument timbres and the change in the induced/perceived color as a result of the partial reduction.

#### 4.1.1 Instrument-dependent color perception

**Figure 1:**
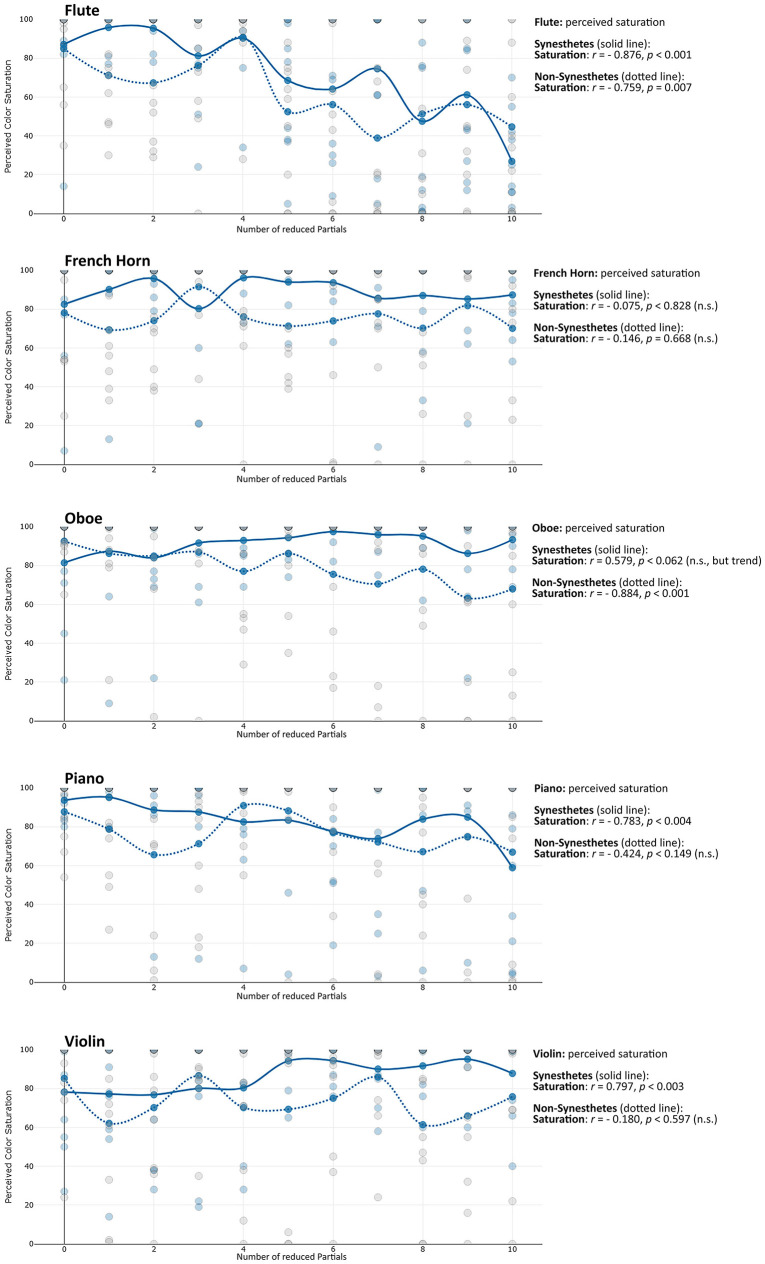
Changes in perceived saturation of the timbre-induced color perception in synesthetes with the number of reduced partials. The individual points reflect the saturation perception of the individual test subjects (blue = synesthetes, grey = non-synesthetes).

**Figure 2:**
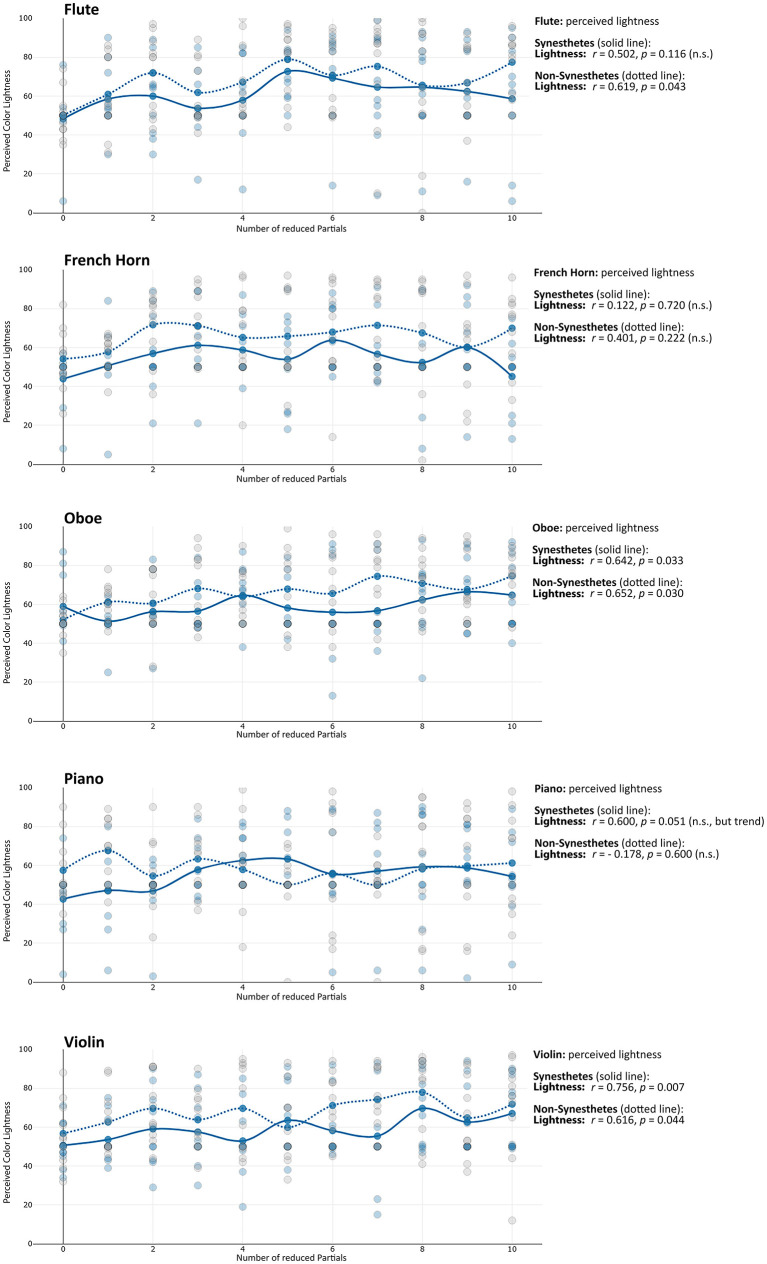
Changes in perceived lightness of the timbre-induced color perception in synesthetes with the number of reduced partials. The individual points reflect the saturation perception of the individual test subjects (blue = synesthetes, grey = non-synesthetes).

**Saturation:** For flute, piano and violin, partial reduction was found to influence the saturation of perceived colors, particularly among synesthetes. As more partials were removed, synesthetes perceived less saturated colors for flute and piano (see Table 2). In contrast, for violin this relationship was reversed: the impression of saturation increased with the number of reduced partials. Among non-synesthetes, a corresponding decrease in saturation was observed for flute and oboe (also see Figure 1). No significant correlations were found for the French horn or for Hue values across any instruments. Overall, the data indicated substantial variability in timbre-induced color saturation across participants, complicating the emergence of a clear overall pattern. For flute and piano (and, among non-synesthetes, for oboe), saturation decreased with the number of reduced partials, meaning that color perception tends to become greyer as more partials were removed. Furthermore, synesthetes and non-synesthetes seem to share the impression of saturation most strongly for the flute. Conversely to these saturation decreases, for violin in synesthetes—and to a lesser extent, oboe in non-synesthetes—the perceived saturation increased with the number of reduced partials (see Figure 2 and Table 2).

**Table 2:** Results of the correlation analysis between the number of reduced partials in relation to the values of the colors perceived by timbre-color synesthetes and non-synesthetes.

	**Flute**	**French horn**	**Oboe**	**Piano**	**Violin**
**Synesthetes**					
Saturation	−0.876^***^	−0.075	0.579	−0.738^**^	0.797^**^
Lightness	0.502	0.122	0.642^*^	0.600	0.756^**^
Red	−0.768^**^	−0.451	−0.050	0.031	0.829^**^
Green	0.401	−0.690^*^	−0.645^*^	0.543	−0.052
Blue	0.788^**^	0.394	0.673^*^	0.588	0.545
**Non-Synesthetes**					
Saturation	−0.759^**^	−0.146	−0.884^***^	−0.424	−0.180
Lightness	0.619^*^	0.401	0.652^*^	−0.178	0.616^*^
Red	0.262	0.093	0.525	−0.387	0.555
Green	0.668^*^	0.706^*^	0.818^**^	0.160	0.468
Blue	0.486	0.249	0.491	−0.491	0.302

Significant results are marked in **blue** for synesthetes and **green** for non-synesthetes (^***^p < 0.001, ^**^p < 0.01, ^*^p < 0.05).

**Figure 3:**
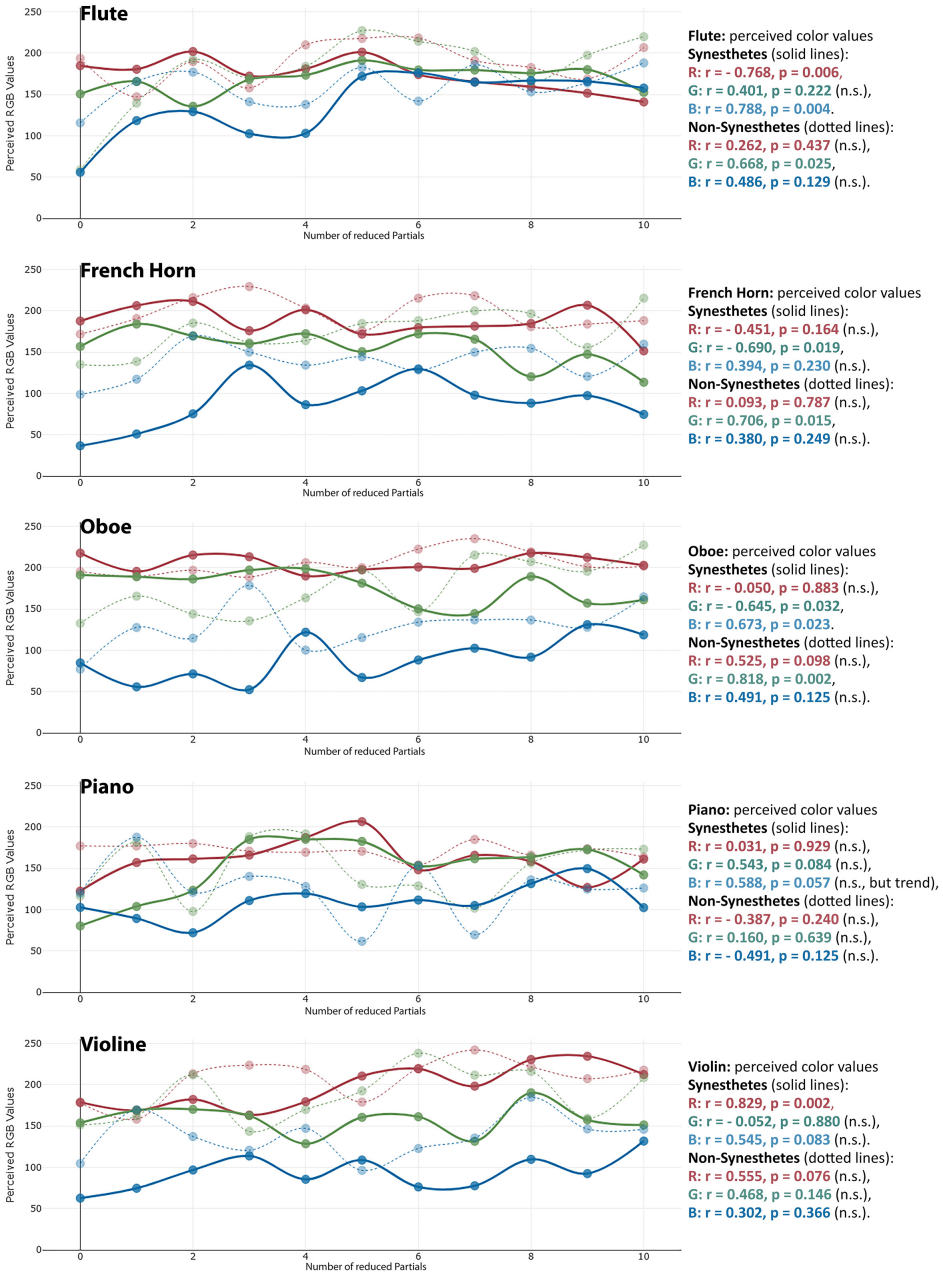
Changes in the perceived RGB values of the timbre-induced color perception in synesthetes with the number of removed partials.

**Lightness:** The findings indicate that an increasing number of reduced partials was generally associated not only with greater timbral brightness but also with greater perceived lightness of the corresponding impressions. This effect was observed particularly for flute (among non-synesthetes), oboe, piano (as a trend), and violin. No such effect was found for the French horn (see Figure 3).

Timbre-induced changes in hue differed between synesthetes and non-synesthetes. Among synesthetes, increasing the number of reduced partials primarily affected the red and blue components (flute, oboe, piano, violin). In contrast, non-synesthetes almost exclusively (and to a comparatively lesser extent) exhibited changes in the green components (flute, French horn, oboe).

**Figure 4:**
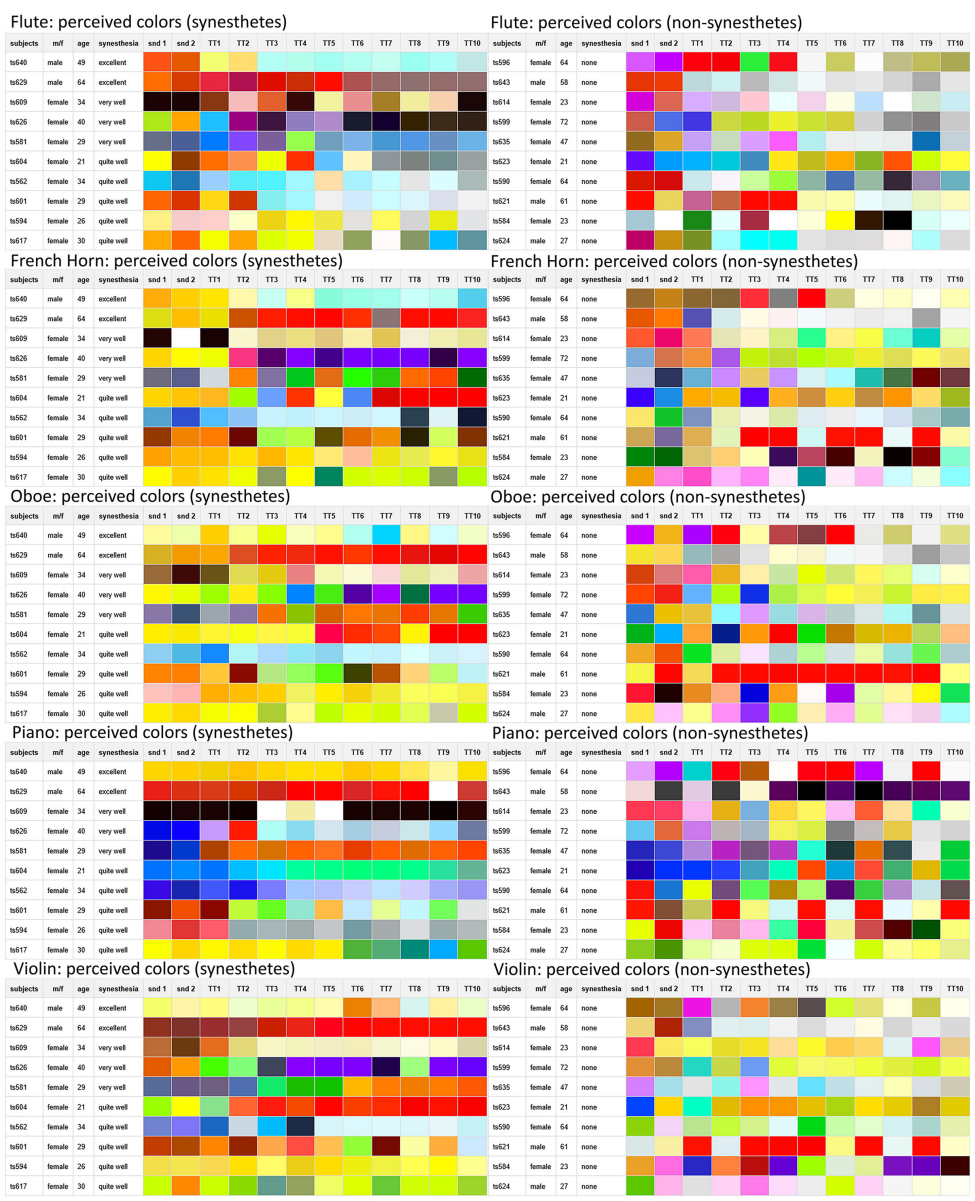
The colors perceived by the synesthetes **(left)** and non-synesthetes **(right)** in comparison, in each case the color perception of the original instrument (twice, snd 1 and snd 2), followed by the partial-reduced sounds from one partial (TT1) up to ten partials (TT10).

**Red:** Red components in colors showed a divergent pattern in synesthetes when comparing the instruments, while red components decreased for flute, they increased for violin and oboe (see Figure 4). This pattern is similar to the connection observed in synesthetes regarding the saturation levels of flute and violin, which was also divergent.

**Green:** While the proportion of green in non-synesthetes also increased with increasing number of reduced partials, it tended to decrease in synesthetes (flute, French horn, oboe).

**Blue:** For synesthetes, however, the blue components of colors increased with the number of removed partials (flute, oboe, piano).

#### 4.1.2 Audio feature-dependent color perception

The timbre-induced color perception found here for instrument timbres arguably appears somewhat inconsistent, even if one takes into account that synesthetic perceptions are always very individual and unique (it is therefore pointless to look for correlations between audio features and the Hue value in the timbre-induced color perception). The picture becomes much clearer when considering specific audio features instead of musical instruments. The individual changes in the synesthetic color perceptions when reducing partials can be linked to a few audio features independent of the instrument.

Since only partials were removed during the reduction process and not any blowing, bowing or striking noises in the corresponding frequency range, it can be assumed that audio features that describe the spectral shape as well as the relationship between tonal/harmonic and percussive components in the spectrum are primarily relevant for the timbre-induced perception of color. Thus, these three audio characteristics should be particularly suitable for a comparative audio signal analysis:

- the Spectral Centroid, as the perceived center of mass of the sound spectrum, indicating its brightness (Peeters et al., 2011),
- the Harmonicity Strength as a presence of fifths, minor and major thirds in the spectrum based on a tone network representation (according to Harte et al., 2006; Czedik-Eysenberg, 2021),
- the Percussive Loudness, as the loudness of the percussive signal components according to ITU-R (2015).

A correlation analysis between color values of the timbre-induced colors (saturation, lightness, as well as red, green, and blue values) and the changing audio properties going along with the partial reduction leads to a revealing table with Pearson's r values, in which person- and instrument-independent patterns in the sound-induced color perception of synesthetes become apparent (see Table 3).

**Table 3:** Results of the correlation analysis between the changes in the audio features (as a consequence of partial reduction) in relation to the values of the colors **perceived by timbre-color synesthetes**.

**Synesthetes**	**Spectral centroid**		**Harmonicity strength**		**Percussive loudness**	
	***r***	***p***	***r***	***p***	***r***	***p***
**Saturation**						
Flute	−0.798^**^	0.003	0.823^**^	0.002	−0.748^**^	0.008
French Horn	−0.023	0.946	−0.053	0.877	0.054	0.875
Oboe	0.591	0.056	−0.695^*^	0.018	0.697^*^	0.017
Piano	−0.713^*^	0.014	0.678^*^	0.022	−0.766^**^	0.006
Violin	0.813^**^	0.002	−0.847^***^	< 0.001	0.795^**^	0.003
**Lightness**						
Flute	0.648^*^	0.031	−0.624^*^	0.040	0.670^*^	0.024
French Horn	0.258	0.443	−0.347	0.296	0.354	0,286
Oboe	0.614^*^	0.044	−0.578	0.062	0.578	0,063
Piano	0.820^**^	0.002	−0.822^**^	0.002	0.772^**^	0.005
Violin	0.755^**^	0.007	−0.632^*^	0.037	0.719^*^	0.013
**Red**						
Flute	−0.641^*^	0.034	0.685^*^	0.020	−0.563	0.071
French Horn	−0.463	0.152	0.418	0.201	−0.419	0.200
Oboe	−0.048	0.888	0.137	0.688	−0.141	0.680
Piano	0.304	0.363	−0.303	0.364	0.255	0.450
Violin	0.784^**^	0.004	−0.733^*^	0.010	0.719^*^	0.013
**Green**						
Flute	0.562	0.072	−0.550	0.079	0.601	0.050
French Horn	−0.630^*^	0.038	0.542	0.085	−0.565	0.070
Oboe	−0.676^*^	0.022	0.677^*^	0.022	−0.640^*^	0.034
Piano	0.784^**^	0.004	−0.806^**^	0.003	0.744^**^	0.009
Violin	−0.070	0.839	0.154	0.651	−0.061	0.858
**Blue**						
Flute	0.852^***^	< 0.001	−0.842^**^	0.001	0.833^**^	0.001
French Horn	0.540	0.086	−0.591	0.055	0.612^*^	0.045
Oboe	0.671^*^	0.024	−0.658^*^	0.028	0.623^*^	0.041
Piano	0.564	0.071	−0.558	0.075	0.558	0.074
Violin	0.599	0.051	−0.483	0.132	0.591	0.055

Significant results are marked in **blue** (^***^p < 0.001, ^**^p < 0.01, ^*^p < 0.05).

Based on these results, the following conclusions can be made about timbre-induced colors for timbre-color synesthetes:

**Saturation:** Although there were a number of strong correlations, these appear to be highly instrument-dependent: For French horn, oboe and violin, timbre-induced color saturation increased with decreasing harmonicity strength and increasing percussive loudness, while it decreased accordingly for flute and piano. In other words, with reduced partials the timbre-induced colors got greyer for French horn, oboe and violin, and more saturated for flute and piano. The possible reasons for this are explained in Section 4.3.1.

**Lightness:** The lightness of the timbre-induced colors seems to depend on the three measured audio features, regardless of the instrument: the higher the spectral centroid and percussive loudness and the lower the harmonicity strength, the brighter the colors induced by the timbre became. In other words, the more partials were removed, the less tonal and brighter the sound got, and the brighter the induced color.

The correlation between RGB values and audio features was not as clear as with saturation and lightness, but still recognizable:

**Red:** Regarding the red component there were only weak correlations or tendencies, which seem to be instrument dependent: The color perceptions induced by flute, oboe and French horn tended to become redder with decreasing spectral centroid, decreasing percussive loudness and increasing harmonicity strength (i.e., the less partials were reduced, the redder the color), whereas with piano and violin the opposite tends to be true (i.e., the more partials were reduced, the redder the color).

**Green:** Similar trends as for saturation could be observed for the green component of the timbre-induced colors: the colors induced by French horn, oboe and violin became greener with decreasing spectral centroid, decreasing percussive loudness and increasing harmonicity strength (i.e., the less partials were reduced, the greener the color), while the opposite effect occurred with piano and flute (i.e., the more partials were reduced, the greener the color).

**Blue:** The blue component of the colors seen by synesthetes showed the same behavior as saturation, but in a slightly weaker way: Here, too, across all instruments the blue component got stronger with higher spectral centroid, increasing percussive loudness and decreasing harmonicity strength (i.e., the more partials were removed, the bluer the induced color became).

Looking at non-synesthetes on the other hand, comparable instrument-independent patterns could also be identified (see Table 4), even if they differed from those of synesthetes. Where lightness and blue components mainly appeared in synesthetes, patterns in non-synesthetes tended to rely on changes in saturation and green components. No significant correlations were found in the blue component range in this group (see Table 4), a trend which was already observed in colors of partial removed sounds (see Table 2).

**Table 4:** Results of the correlation analysis between the changes in the audio features (as a consequence of partial reduction) in relation to the values of the colors **perceived by non-synesthetes**.

**Non-synesthetes**	**Spectral centroid**		**Harmonicity strength**		**Percussive loudness**	
	***r***	***p***	***r***	***p***	***r***	***p***
**Saturation**					
Flute	−0.758^**^	0.007	0.763^**^	0.006	−0.695^*^	0.018
French Horn	−0.059	0.863	0.023	0.947	−0.040	0.907
Oboe	−0.865^***^	< 0.001	0.864^***^	< 0.001	−0.852^***^	< 0.001
Piano	−0.266	0.430	0.268	0.425	−0.361	0.275
Violin	−0.164	0.630	0.078	0.820	−0.185	0.586
**Lightness**					
Flute	0.682^*^	0.021	−0.637^*^	0.035	0.712^*^	0.014
French Horn	0.479	0.136	−0.442	0.174	0.536	0.089
Oboe	0.825^**^	0.002	−0.822^**^	0.002	0.881^***^	< 0.001
Piano	−0.382	0,247	0.355	0.284	−0.259	0.442
Violin	0.623^*^	0.040	−0.599	0.052	0.643^*^	0.033
**Red**					
Flute	0.328	0,325	−0.326	0.328	0.373	0.258
French Horn	−0.007	0,983	−0.015	0.965	0.062	0.857
Oboe	0.586	0,058	−0.624^*^	0.040	0.607^*^	0.047
Piano	−0.434	0,182	0.466	0.149	−0.456	0.158
Violin	0.617^*^	0.043	−0.651^*^	0.030	0.641^*^	0.033
**Green**					
Flute	0.755^**^	0.007	−0.703^*^	0.016	0.800^**^	0.003
French Horn	0.709^*^	0.015	−0.639^*^	0.034	0.710^*^	0.014
Oboe	0.838^**^	0.001	−0.743^**^	0.009	0.818^**^	0.002
Piano	0.107	0.755	−0.130	0.704	0.194	0.567
Violin	0.481	0.134	−0.554	0.077	0.509	0.110
**Blue**					
Flute	0.463	0.151	−0.431	0.185	0.439	0.177
French Horn	0.421	0.197	−0.354	0.285	0.472	0.143
Oboe	0.441	0.175	−0.474	0.141	0.489	0.127
Piano	−0.371	0.261	0.316	0.344	−0.297	0.375
Violin	0.229	0.499	−0.067	0.844	0.213	0.530

Significant results are marked in **green** (^***^p < 0.001, ^**^p < 0.01, ^*^p < 0.05).

Even though there were overall fewer and weaker correlations in the color perception of non-synesthetes, the following conclusions can be made:

**Saturation:** Similar consistent patterns as the ones in synesthetes emerge in terms of saturation, where the lower the spectral centroid and the percussive loudness and the stronger the harmonicity strength—i.e., the less partials are reduced—the more saturated the color selected is.

**Green:** The higher the spectral centroid and the percussive loudness and the weaker the harmonicity strength—i.e., the more partials are reduced—the greener the color impression becomes.

In other words, as additional partials are removed from the sound, non-synesthetes perceive the resulting color impression as not only becoming greener but also greyer, whereas for timbre-color synesthetes, the timbre-induced color becomes lighter and more bluish.

#### 4.1.3 Summarized key differences of synesthetes and non-synesthetes

**Synesthetes:** Timbre-induced color changes are highly instrument-dependent and exhibit the strongest correlations with lightness and blue components. As partials are removed:

1. Saturation increases for some instruments (for flute and piano) while it decreases for others (French horn, oboe, and violin) and shows overall more variable changes than other features.
2. Lightness and blue components clearly increase with reduced partials for most instruments.
3. Red components increase for some instruments (piano and violin) while decrease for others (mostly flute and French horn). Green components increase for piano and flute and decrease for French horn and oboe. Blue components increase for all instruments.

**Non-Synesthetes:** Timbre-induced color changes are more instrument-independent and interestingly, blue components play no significant role at all. As partials are removed:

1. Saturation of colors decreases (noticeable for flute and oboe); for the flute this trend is contrary to synesthetes.
2. Lightness changes are less pronounced and not clearly tied to specific audio features but can be summarized for flute and oboe.
3. Green components increase (for flute, French horn and oboe). Red components play only a minor role.

### 4.2 Timbre-induced color perception with morphed sounds

Similar results to those obtained with the reduced partials are also evident when the original sounds are gradually morphed with ratios of 10:90, 30:70, 50:50, 70:30, and 90:10.

In order to investigate whether and to what extent the perceived colors of the sounds morphed in these 5 ratios or stages deviate from the induced colors of the original sounds, the color differences between the morphing stages and the original sound were calculated using the CIEDE2000 algorithm (Cui et al., 2001). Since the color perceptions induced by the original sound were recorded twice (see above), in many cases there were two not quite identical starting colors and two not quite identical ending colors, so that 2 × 2 color distance curves were considered for each morphing process. With 5 instrument sounds that could be morphed with each other in two directions, a total of 20 different morph combinations with 2 color distance curves each took place in the 5 different morphing ratios (see Table 5).

**Table 5:** Combinations of instruments morphed with each other, each with two color distance curves, which are shown below with either **red** and **green** or **orange** and **blue** trend lines per chart (see Figure 5).

	**Flute**		**Violin**		**French Horn**		**Piano**		**Oboe**	
Flute										
Violin										
French Horn										
Piano										
Oboe										

**Figure 5:**
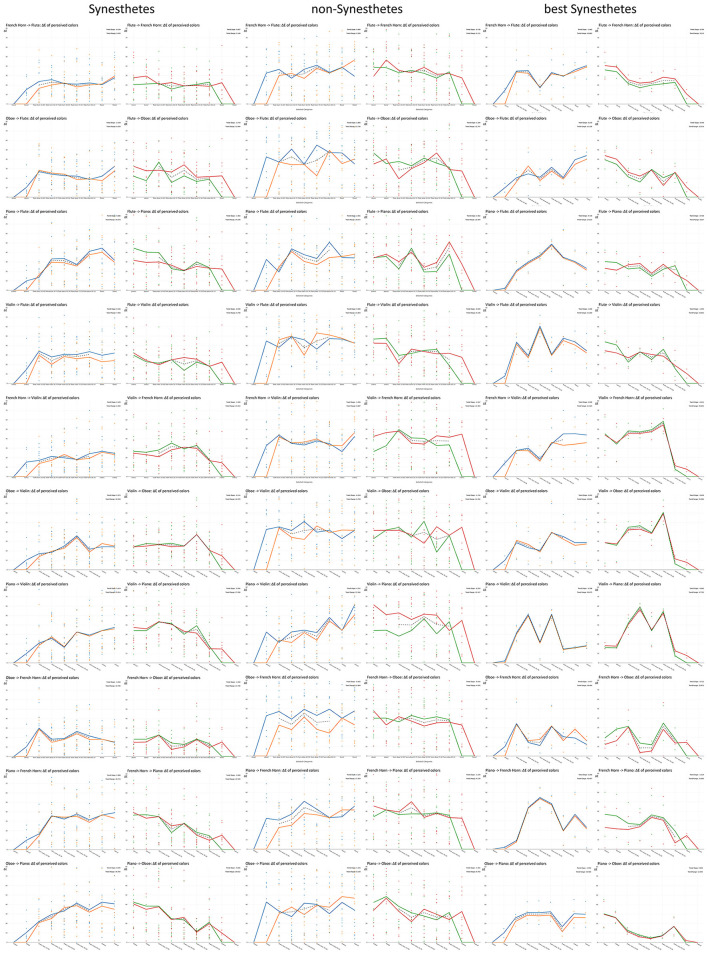
Calculated color differences (in Δ*E* on the *Y*-axis) to the induced colors of the initial sound, measured twice in each case, and their trend lines in synesthetes **(column left)**, non-synesthetes **(column center)** and the two strongest synesthetes **(column right)**.

The trend lines generated from the scatter plots of all calculated color distances—organized by synesthetes, non-synesthetes, and the two best synesthetes—revealed remarkable differences between these three groups:

1. The color distance between the original sounds and the first morph stage was noticeably greater for non-synesthetes (they had a noticeably greater slope in the trend line) than for synesthetes (i.e., for non-synesthetes, the colors induced by the morphed sounds were far less related to the original colors, than for synesthetes).
2. It can be seen that the two best synesthetes experienced much greater color differences on average than the synesthetes as a whole. Also, the synesthetes in turn experienced greater differences than the non-synesthetes (this can of course be explained by the fact that the trend curves already reflect the average color distance, which is more leveled out with 10 people than with 2, yet the color distances among synesthetes are more uniform than those among non-synesthetes). In many cases, the two best synesthetes showed a distinctive dip in the color distance in the middle of the curve (at a 50:50 morph), which suggests that a new instrument = a new color was perceived here. Also noteworthy here is the consistency in which the color distances of the morphed sounds to the colors of the original sounds were perceived by the two best synesthetes. This result is also reflected in the slopes and ranges of the individual trend lines (see Figure 5).

While the overall slopes of the trend lines suggest that the distances between the colors of the original sounds and those of the morph sounds increased equally in synesthetes and non-synesthetes, the more the morphed sound deviated from the original sound (Table 6, mean of the left columns), a differentiated analysis of only the morphed sounds showed that the color distances among synesthetes had much clearer or steeper gradients than among non-synesthetes (Table 6, mean of the right columns). Furthermore, looking at the Δ*E* ranges in which the trend lines move, it can be seen that the trend lines range here were much larger for non-synesthetes than for synesthetes (Table 7, mean of the left columns). However, when looking only at the trend lines for the color distances between the morphed sounds and the original sounds, it becomes apparent that the ranges for non-synesthetes were small compared to those for synesthetes and, above all, for best synesthetes.

**Table 6:** Slope of the trend lines for the individual morph combinations, for synesthetes, non-synesthetes, and best synesthetes, both in their totality (**left**) and only for the morphed sounds (**right**).

	**Trend slopes: overall**			**Trend slopes: morphed sounds only**		
	**Synesth**.	**Non-synesth**.	**Best synesth**.	**Synesth**.	**Non-synesth**.	**Best synesth**.
Fr.Horn → Flute	2.546	3.789	3.879	−0.139	0.906	−1.065
Flute → Fr.Horn	2.311	3.824	4.001	−0.007	0.738	−0.769
Oboe → Flute	2.388	3.814	4.473	−2.154	1.856	0.370
Flute → Oboe	2.847	3.708	4.366	2.714	−1.339	−0.168
Piano → Flute	4.643	3.846	3.710	4.386	2.931	4.718
Flute → Piano	4.049	3.136	2.913	1.963	−1.092	0.738
Flute → Violin	2.859	4.235	4.316	0.156	0.930	1.006
Violin → Flute	2.776	4.550	4.437	0.559	0.004	1.550
Fr.Horn → Violin	2.713	3.389	4.787	1.129	−1.446	2.958
Violin → Fr.Horn	2.814	3.536	5.608	1.496	2.267	6.421
Oboe → Violin	3.115	3.536	3.344	2.572	−0.380	2.202
Violin → Oboe	2.590	3.748	3.944	0.111	1.744	5.409
Piano → Violin	4.311	5.447	1.400	2.414	4.761	−3.308
Violin → Piano	4.772	4.253	2.906	5.991	0.283	6.660
Oboe → Fr.Horn	1.489	3.563	1.645	−1.062	−0.392	−1.477
Fr.Horn → Oboe	1.817	4.015	1.925	1.500	0.516	0.722
Piano → Fr.Horn	4.500	4.339	3.664	2.988	2.125	2.590
Fr.Horn → Piano	4.727	4.128	3.305	4.984	1.239	1.514
Piano → Oboe	4.889	4.087	2.711	3.649	1.193	−1.954
Oboe → Piano	5.161	4.347	3.110	4.845	1.481	−0.948
**Mean**	**3.366**	**3.965**	**3.522**	**1.905**	**0.916**	**1.358**

**Table 7:** Range of the trend lines for the individual morph combinations, for synesthetes, non-synesthetes, and best synesthetes, both in their totality (**left**) and only for the morphed sounds (**right**).

**Overall ranges**	**Trend ranges: overall**			**Trend ranges: morphed sounds only**		
	**Synesth**.	**Non-synesth**.	**Best synesth**.	**Synesth**.	**Non-synesth**.	**Best synesth**.
Fr.Horn → Flute	28.645	39.336	39.922	3.435	9.538	16.883
Flute → Fr.Horn	25.368	42.476	38.333	2.194	6.131	5.215
Oboe → Flute	30.237	48.325	42.058	8.539	13.730	11.108
Flute → Oboe	32.797	41.357	41.755	13.949	12.747	12.518
Piano → Flute	42.427	42.734	48.168	24.378	20.671	27.810
Flute → Piano	38.120	44.794	26.793	14.194	22.954	9.627
Flute → Violin	32.072	49.821	59.523	7.955	11.824	31.260
Violin → Flute	31.008	45.127	39.267	6.758	9.106	15.832
Fr.Horn → Violin	26.248	44.552	39.963	6.300	8.887	21.625
Violin → Fr.Horn	31.860	48.870	56.575	14.410	11.464	46.843
Oboe → Violin	34.972	44.694	39.417	19.549	6.794	19.800
Violin → Oboe	37.136	43.270	59.980	16.379	11.232	51.090
Piano → Violin	36.770	56.225	50.992	15.914	23.346	36.675
Violin → Piano	43.477	49.071	57.915	27.030	10.167	47.730
Oboe → Fr.Horn	29.483	45.332	33.850	12.796	11.864	19.460
Fr.Horn → Oboe	22.643	43.867	31.543	11.736	4.455	22.875
Piano → Fr.Horn	36.802	44.186	54.017	20.715	17.324	45.457
Fr.Horn → Piano	37.849	43.773	34.935	22.529	9.139	21.995
Piano → Oboe	40.409	45.311	30.678	18.704	8.134	16.580
Oboe → Piano	41.563	47.572	30.082	26.812	8.783	12.548
**Mean**	**33.9943**	**45.53465**	**42.7883**	**14.7138**	**11.9145**	**24.64655**

#### 4.2.1 Audio feature-dependent color perception

The timbral changes associated with the various morphing stages or ratios can again be described in terms of numbers for the individual audio features with the help of audio signal analysis. Here, too, a similar pattern emerged as with the partial-reduced sounds (see above), namely that changes in the perceived color values could be traced back primarily to the three previously used audio features: The spectral centroid, the harmonicity strength and the percussive loudness as audio features that primarily relate to the shape, harmonic and percussive components of the spectrum have again proven to be significant sound characteristics for describing the observed changes. And as in the study with partial-reduced sounds, it was again the proportion of percussive, noisy components that was particularly influential for the color perception of synesthetes:

With regard to hue and color saturation, no meaningful correlations with the changing timbral features could be found; not for color hue, because it is measured in degrees and varies greatly from person to person; and not for color saturation, because, in contrast to the study with partial-reduced timbres, no general tendency for a change in color saturation depending on the morph level was apparent here. However, clear correlations were found between timbral changes and the lightness of the induced colors, expressed in the same way in both synesthetes (see Table 8) and non-synesthetes (see Table 9) as well as in red, green, and blue values with slight differences between the two groups.

**Table 8:** Results of the Bonferroni-corrected (*n* = 3 ^*^ 5 = 15) correlation analysis between the changes in the audio features (as a consequence of the morphing stage) in relation to the values of the colors **perceived by timbre-color synesthetes**.

**Synesthetes**	**Spectral centroid**	**Harmonicity strength**	**Percussive loudness**
Saturation	−0.182	0.121	0.049
Lightness	0.627^***^	−0.616^***^	−0.056
Red	0.510^*^	−0.500^**^	−0.496^**^
Green	0.429^*^	−0.388^*^	−0.330
Blue	0.144	−0.146	0.449^*^

Significant results are marked in **blue** (^***^p < 0.000066, ^**^p < 0.00066, ^*^p < 0.0033).

**Table 9:** Results of the Bonferroni-corrected (*n* = 3 ^*^ 5 = 15) correlation analysis between the changes in the audio features (as a consequence of the morphing stage) in relation to the values of the colors **perceived by non-synesthetes**.

**Non-synesthetes**	**Spectral centroid**	**Harmonicity strength**	**Percussive loudness**
Saturation	−0.248	0.143	−0.233
Lightness	0.675^***^	−0.558^***^	0.122
Red	0.400^*^	−0.309	−0.108
Green	0.634^***^	−0.538^***^	−0.017
Blue	0.164	−0.159	0.090

Significant results are marked in **green** (^***^p < 0.000066, ^**^p < 0.00066, ^*^p < 0.0033).

These correlations allowed the following overall statements to be made about the color perception induced in timbre-color synesthetes by timbral characteristics.

**Lightness:** The lightness of the timbre-induced colors seemed to be dependent on spectral centroid and the harmonicity strength (for both synesthetes and non-synesthetes): The higher the spectral centroid (i.e., the brighter the timbre) and the fewer harmonic components the sound had, the brighter the color induced by it would be.

**Red:** The higher the spectral centroid (i.e., the brighter the timbre), the lower the percussive and harmonic components in the sound, the stronger the red components in the sound-induced color perception.

**Green:** For the green values, similar principles applied as for the red values, only less pronounced.

**Blue:** Here, the loudness of the percussive components in the sound was the decisive factor: the more percussive or noisy the sound, the stronger the blue components in the sound-induced color perception were.

**Figure 6:**
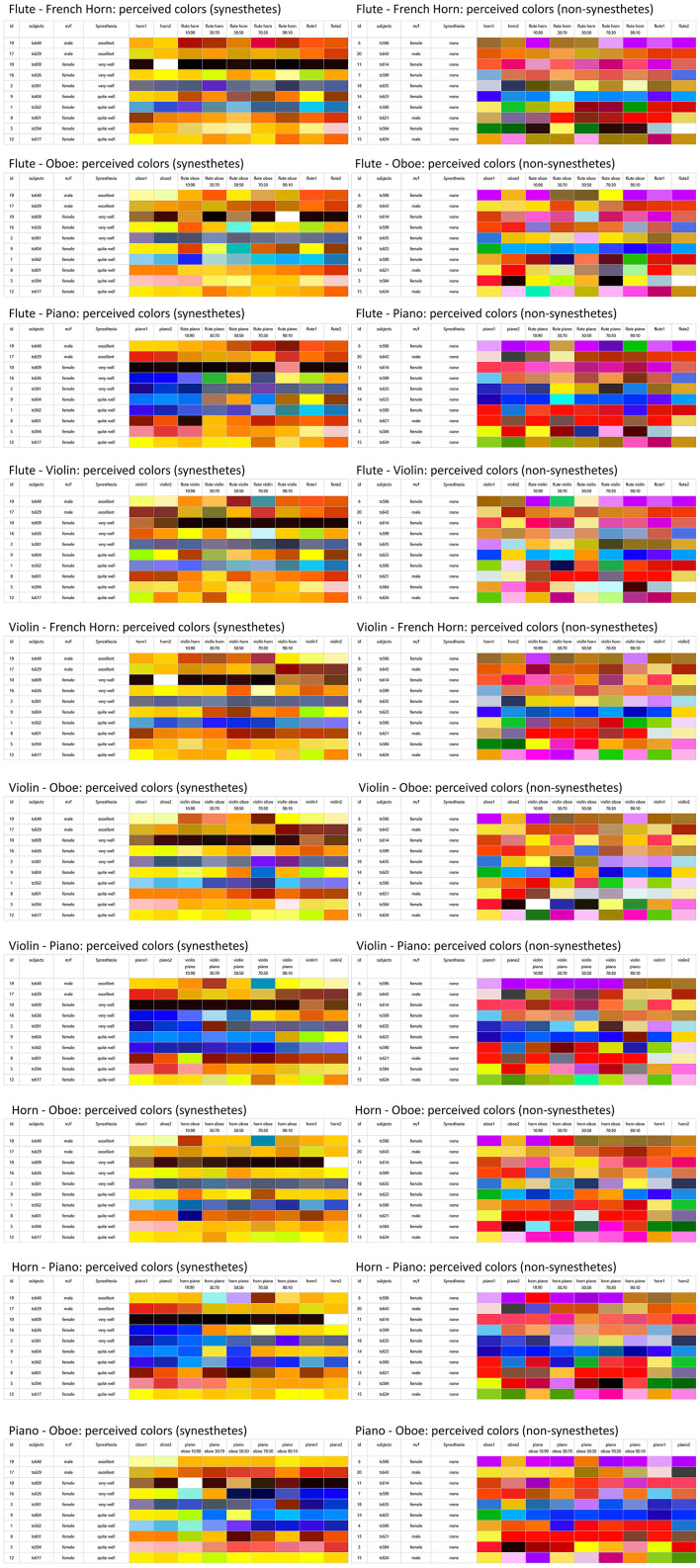
The colors perceived by the synesthetes **(left)** and non-synesthetes **(right)** in comparison, in each case the color perception of the original instrument (twice, snd 1 and snd 2), followed by the morphed sounds in the ratios 10:90, 30:70, 50:50, 70:30, 90:10 and followed by the target the original instrument.

Here, too, the effect already observed before can be seen: The imagined color in non-synesthetes was particularly influenced by the green values, while in synesthetes it was the peripheral color ranges, i.e., the red and blue values, that were particularly sensitive to timbral changes. In the blue values in particular, the percussive components in the sound played a major role. This was also reflected in the colors that synesthetes have assigned to the individual musical instrument timbres and their morphs: colors with strong blue components were particularly prevalent in the percussive piano sound and its morph derivatives (see Figure 6).

## 5 Discussion

The high amount of induced blue color perceptions for piano sounds was also observed in a previous study on timbre-color synesthetes (Orlandatou, 2014), although in that study, the color perception was thought to be more due to the sad character of the piano piece [L.v. Beethoven Adagio sostenuto (1st part), Piano Sonata No. 14, op. 27, No. 2 in C-sharp minor]. The results of our morph study imply that the blue components in sound-induced color perception can be attributed to the percussive elements typical of the beginning of the piano sound.

If the colors that synesthetes and non-synesthetes perceived for the individual sounds are arranged in an instrument-dependent order (Figures 4, 6), it can be seen that the synesthetes' perception of colors for timbres with reduced partials is far more consistent and structured than that of non-synesthetes (similar in Ward et al., 2006). However, it is certainly not the case that consistent color sequences can be always observed in synesthetes: There seem to be instruments that are more easily assigned to a consistent color by synesthetes, such as the piano in this case, while the successive colors in reduced partials are far less coherent or consistent in the case of the oboe or horn. Similar effects can also be observed in grapheme-color synesthesia (e.g., Ward, 2019; Lacey et al., 2021), which also shows strong deviations in the strength and consistency of color perception for letters and numbers.

In case of the partial-reduced sounds another striking feature can be observed, which is that the perceived color of synesthetes changes fundamentally in many cases (mostly to the opposite color) after the second partial is removed. This may be related to the fact that the timbre changes noticeable with the removal of the 2nd partial (octave), since the 3rd partial (fifth above the octave) then suddenly takes on a stronger significance in the perceived timbre (since it is no longer octave-equivalent).

**Figure 7:**
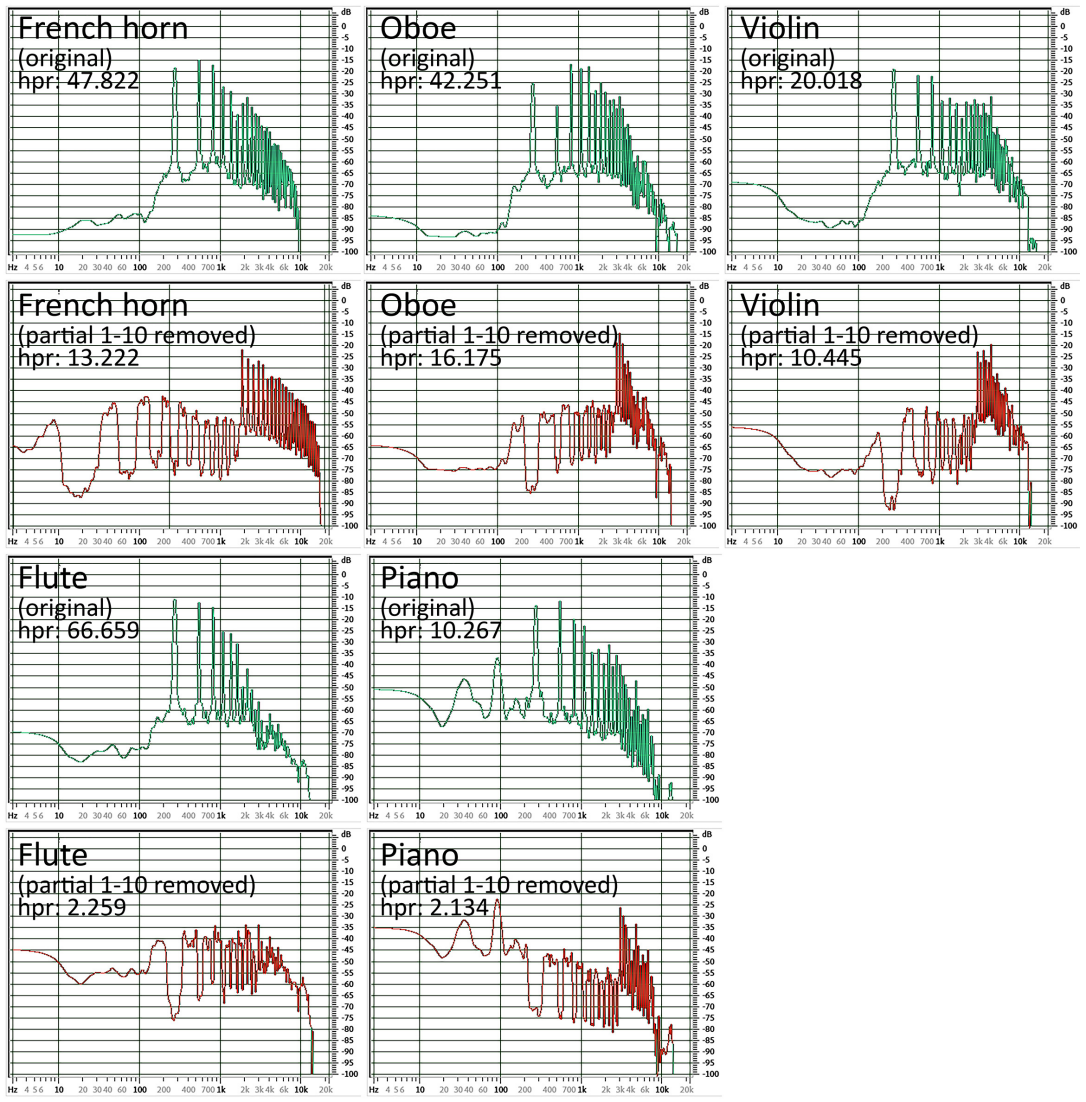
Comparison of the mean spectra and their harmonic percussive ratio (hpr) of the original timbres (green spectra) and the timbres after the reduction of the first 10 partials (red spectra): Even after removing the first 10 partials, the tonal components of French horn, oboe and violin remain loud enough (hpr > 10) to be stronger than the respective noise components of the instrument. This is not the case with flute and piano, here the amplitudes of the remaining partials are too low (hpr < 3) to give the sound a sufficiently strong pitch information. The sound is perceived more as noise, which in many cases leads to an unsaturated color perception for the synesthetes.

In addition, it is noticeable that for flute and piano, the perceived colors become increasingly greyer with increasing partial reduction for many test subjects, which is also reflected in the decreasing saturation of the timbre-induced colors when the higher partials are reduced (see above, compare Figure 1 with Figure 4). The question arises as to why the decrease in the saturation of the tonally induced colors with increasing partial reduction can be observed particularly in the flute and piano, while the saturation remains almost unchanged in the horn, oboe and gets even stronger in case of the violin (see Table 2, line “saturation”). One possible reason could be the relationship between harmonic and percussive components already observed in the audio signal analysis [this harmonic-percussive ratio (hpr, based on harmonic-percussive source separation, Driedger et al., 2014) has a strong correlation to the measured percussive loudness (see above) of *r* = −0.830, *p* < 0.001]: The ratio between tonal and noise components in the original sound of the French horn and oboe is already much lower than that in the piano (attack noise) and flute (blowing noise). If more and more partials are removed from the spectrum while the noise components in the same frequency range still remain the same, the proportion of noise increases substantially with each partial removed, especially when considering that the stimuli presented here are loudness-matched. The question remains as to why the violin, with its strong noise components (bowing noise), does not also show a decrease in saturation in the tonally induced colors: Although the violin also has a lot of background noise in its original timbre, the tonal components above the 10th partial are still strong enough to create a clear pitch impression. They are much stronger than the lower frequency noise components, whereas with the piano and flute, as the partial reduction increases, the remaining high partials are no longer sufficient to counterbalance the strong lower frequency noise components (see Figure 7).

### 5.1 Limitations

Despite the promising and interpretable results, this pilot study has several limitations and leaves open questions that should be addressed in future research.

*Which colors are real?* Timbre-color synesthetes do not typically experience sound-induced percepts as a single, discrete color that can be directly mapped to an RGB or HSL value. Instead, they often perceive dynamic and complex structures, such as “the sound is dark, like a dark brown nearly black then going into yellow” (test subject ts629, describing a timbre morphed in a 50:50 ratio between French horn and piano). When synesthetes are required to select only one color per sound, these complex color perceptions are inevitably reduced during data collection.

*Which synesthetes are real?* Of the 20 timbre-color synesthetes originally recruited, only half (10 individuals) met the predefined criteria for confirmed synesthesia, demonstrating satisfactory or greater consistency in their color perceptions. The remaining 10 were considered “non-synesthetes” for the purposes of further evaluation. This division could limit the generalizability of the findings, particularly with regard to non-synesthete responses. There is a legitimate question as to the extent to which the group of “non-synesthetes” genuinely consists of non-synesthetes, rather than of synesthetes who simply did not perform well enough in the task assigned to them here (“failed synesthetes”). This is especially pertinent when considering that synesthesia can occur in varying degrees of intensity. For example, despite an average Δ*E* of 19.358 and 20.808, respectively, the participants with the Nos. 596 and 643 could still be classified with certainty as “failed synesthetes”, especially since they exhibit high color consistency on three of the five instruments. However, among the other subjects classified as “non-synesthetes”, there were only two, one, or even no consistent color perceptions, which makes it increasingly difficult to classify them as “failed synesthetes”. Thus, when it comes to the group of non-synesthetes it remains unclear if they are non-synesthetes in a strict sense. One can only say that these are people who, on average, experienced a different color (Δ*E* > 16) rather than a matching/similar color (Δ*E* < 16) for the same sound.

*Which pitch is the right one?* All stimuli in this study were presented at a single pitch (C4). However, prior research has shown that different pitches can elicit distinct color perceptions in synesthetes (Glasser, 2018, 2023; Itoh et al., 2017; Ward et al., 2025). These pitch-dependent differences were not incorporated into the present design and therefore remain an open question for future work.

*Which dependencies between the stimuli should be considered?* The Pearson correlation used here is based on the assumption that each observation (i.e., the combination of a specific sound and the color it induces) is independent of the other observations. In practice, however, because the sounds were altered by a systematic reduction of the morph ratios, the independence of the stimuli from one another can be questioned. Within a sequence of morphed timbres, audio features (and the resulting color properties) are likely to correlate more strongly with each other (e.g., a 90:10 flute-oboe timbre is very similar to the original flute timbre, which could lead to similar audio features and similar color perceptions). This could result in an underestimation of the significance of the correlation (*p*-value) and an overestimation of the strength of the correlation (*r* value). Therefore, this increased risk of an alpha error—a correlation appears significant even though it is not—should be taken into account, although the results presented here already apply a strict significance level to the values determined, using a Bonferroni correction of *n* = 15 in the second study (morphed sounds), respectively. Furthermore, instrument-specific timbre differences (e.g., blowing or bowing noises) and differences resulting from morphing may influence the results of the correlation analysis, as they cannot be measured completely separately from each other. For this reason, the first study (partial-reduced timbres) evaluated each musical instrument separately, but this was not possible in the second study (morphed timbres), where no single instrument could be considered in isolation.

*What would Carlo Emilio Bonferroni say?* To be strict, a Bonferroni correction should be applied in the first study (partial-reduced timbres) based on the number of audio features (3) times the number of color values examined (5) times the number of instruments considered (5) (i.e., *n* = 3 ^*^ 5 ^*^ 5 = 75). With a resulting significance threshold of *p* < 0.000667, this would mean that only an extremely small number of correlations could be considered significant. For synesthetes: the greater the harmonicity strength of the violin timbre, the lower the saturation of the timbre-induced color (*r* = −0.847), and the higher the spectral centroid of the flute, the more blue components in the perceived color (*r* = 0.852). For non-synesthetes in the case of the oboe: the lower the spectral centroid (*r* = −0.865) and the percussive loudness (*r* = 0.852) and the stronger the harmonicity strength (*r* = 0.864), the higher the saturation, and the higher the percussive loudness (*r* = 0.881), the higher the lightness of the perceived color.^2^ Since this is an initial pilot study on the use of audio signal analysis to detect audio features that have a systematic influence on the timbre-induced color perception, we decided not to set an overly strict significance threshold in favor of a more informative compilation of the color-shaping influence of these audio features. Since the three audio features used in the two studies presented here led to comparable and plausible results, it makes sense to examine these in more detail in follow-up studies, limiting the selection of stimuli and color values examined in favor of a convenient Bonferroni correction.

## 6 Conclusion

This study examined the timbre-induced color perception of synesthetes using two approaches: the gradual reduction of partials and the morphing of instrument timbres. The findings reveal clear, though highly individual, patterns of color perception among timbre-color synesthetes, which differ significantly from those observed in non-synesthetes.

A reduction in partials from the fundamental to the 10th partial was associated with an increase in lightness in the color perceptions of timbre-color synesthetes. Timbre-induced colors tended to become progressively lighter with increasing percussive loudness and spectral centroid and decreasing harmonicity strength. Moreover, timbre-induced colors exhibited specific dependencies on the red and blue components. The presence of a greater number of harmonic components in the sound spectrum was associated with stronger red components in the perceived colors, whereas a higher proportion of noisy or percussive components corresponded to stronger blue components. These patterns also extend to color perception in morphed sounds: across morph stages, the perceived colors gradually shifted further away from those induced by the original timbre and closer and closer to the color induced by the target. Interestingly, synesthetes with particularly pronounced timbre-color synesthesia often reported a distinct new color at a 50:50 morph ratio that deviates strongly from the other colors of the sound combination, suggesting the perceptual emergence of an entirely novel sound. This could be interpreted as an expression of the hyperconnectivity-hyperbinding model (Zamm et al., 2013), which suggests that a hybridized stimulus (50:50 morph) is perceived not only as a transition but as a qualitatively new entity with a new, consistent synesthetic correlate, due to a locally particularly enhanced accumulation of white matter in the right inferior fronto-occipital fasciculus as a connection between the occipital lobe (visual association areas) and the temporal lobe (auditory association areas) with the frontal lobe.

Together, these findings underscore the intricate and systematic interplay between auditory and visual modalities in the synesthetic brain. By revealing how spectral transformations shape the phenomenology of color perception, this study provides a basis for future research bridging psychoacoustics, neuroscience, and the phenomenology of musical experience.

## Data Availability

The datasets presented in this study including all stimuli and interactive diagrams are available at https://muwiserver.univie.ac.at/synesthesia/.

## Footnotes

1 You can find all timbre-induced color impressions of the test subjects to the original instruments in the supplementary material at https://muwiserver.univie.ac.at/synesthesia/.

2 See the respective Tables 3 and 4 in a Bonferroni-corrected version in the online supplement at https://muwiserver.univie.ac.at/synesthesia/bonferroni_corrected_tables.pdf.
